# Sialic acid removal by *trans*-sialidase modulates MMP-2 activity during *Trypanosoma cruzi* infection

**DOI:** 10.1016/j.biochi.2021.04.005

**Published:** 2021-04-20

**Authors:** Daniel Musikant, Romina Higa, Cristina E. Rodríguez, Martin M. Edreira, Oscar Campetella, Alicia Jawerbaum, María S. Leguizamón

**Affiliations:** a Departamento de Química Biológica, Facultad de Ciencias Exactas y Naturales, Universidad de Buenos Aires, Intendente Güiraldes 2160, C1428EGA, Ciudad de Buenos Aires, Argentina; b Consejo Nacional de Investigaciones Científicas y Técnicas, (CONICET) Godoy Cruz 2290, C1425FQB, Ciudad de Buenos Aires, Argentina; c Laboratorio de Reproducción y Metabolismo, CEFYBO-CONICET, Facultad de Medicina, Universidad de Buenos Aires, Paraguay 2155 C1121ABG, Ciudad de Buenos Aires, Argentina; d Departamento de Microbiología, IMPAM-CONICET, Facultad de Medicina, Universidad de Buenos Aires, Paraguay 2155 C1121ABG, Ciudad de Buenos Aires, Argentina; e Instituto de Química Biológica de la Facultad de Ciencias Exactas y Naturales IQUIBICEN-CONICET, Universidad de Buenos Aires, Intendente Güiraldes 2160 C1428EGA, Ciudad de Buenos Aires, Argentina; f Instituto de Investigaciones Biotecnológicas IIBio, Universidad Nacional de San Martín, 25 de Mayo y Francia B1650HMP, San Martín, Provincia de Buenos Aires, Argentina

**Keywords:** Chagas disease, Neuraminidase, Matrix metalloproteinases, Neuraminic acid

## Abstract

Matrix metalloproteinases (MMPs) not only play a relevant role in homeostatic processes but are also involved in several pathological mechanisms associated with infectious diseases. As their clinical relevance in Chagas disease has recently been highlighted, we studied the modulation of circulating MMPs by *Trypanosoma cruzi* infection. We found that virulent parasites from Discrete Typing Units (DTU) VI induced higher proMMP-2 and MMP-2 activity in blood, whereas both low (DTU I) and high virulence parasites induced a significant decrease in proMMP-9 plasma activity. Moreover, *trans*-sialidase, a relevant *T. cruzi* virulence factor, is involved in MMP-2 activity modulation both *in vivo* and *in vitro.* It removes α2,3-linked sialyl residues from cell surface glycoconjugates, which then triggers the PKC/MEK/ERK signaling pathway. Additionally, bacterial sialidases specific for this sialyl residue linkage displayed similar MMP modulation profiles and triggered the same signaling pathways. This novel pathogenic mechanism, dependent on sialic acid removal by the neuraminidase activity of *trans*-sialidase, can be exploited by different pathogens expressing sialidases with similar specificity. Thus, here we present a new pathogen strategy through the regulation of the MMP network.

## 1. Introduction

Chagas disease is a chronic disabling infection endemic to Latin America caused by the protozoan *Trypanosoma cruzi.* There is an estimated 6 million infected people and an alarming 50,000 deaths/year with 65–100 million people at risk for infection worldwide. Migration of infected people to developed countries made this regional issue into a global one because of congenital and blood transmission [1].

Once the acute phase is solved patients enter an asymptomatic chronic phase that, after 20–30 years, leads to clinical manifestations such as cardiomyopathy and/or megaviscera in 30–40% of cases. Cardiac parasite persistence induces an inflammatory and fibrotic process that causes intense tissue remodeling, generating cardiomegalia and functional alterations, which can then lead to death [1]. However, the mechanisms involved in this complex scenario remain to be completely understood.

Matrix Metalloproteinases (MMPs) are Zn^2+^-dependent endopeptidases that degrade extracellular matrix proteins, playing an important physiological role in immunomodulation events and in tissue repair and remodeling [2]. They are usually secreted as inactive pro-MMPs which are cleaved to form the active MMP. During inflammatory processes they regulate leukocyte migration through modulation of cytokine and chemokine gradients and degrade physical barriers [2]. MMPs imbalance has been associated with development of pathologies in chronic infectious diseases, thus MMPs have been proposed as therapeutic targets [3,4]. Specifically, MMP-2 and MMP-9, also known as gelatinases, are expressed in various cell-types and are involved in angiogenesis, vascular disease, tumor progression, inflammation, etc. [2]. In Chagas disease, circulating MMP-2 and -9 were proposed as biomarkers for asymptomatic to cardiac form progression [5–7], and alterations in their activity and expression during *T. cruzi* acute experimental infection have also been observed [8–11].

Among the different parasite molecules described as *T. cruzi* virulence factors, *trans*-sialidase (TS) plays several different roles in pathogenesis and its activity is essential to parasite survival [12]. TS belongs to a gene family of about 1430 members that are distributed in eight groups [13]. One of them contains genes encoding active enzyme (aTS), characterized by a Tyr_342_ residue crucial for enzyme activity [14], and other genes encoding the enzymatically inactive proteins (iTS) containing a Tyr_342_His mutation instead. As *T. cruzi* is unable to synthetize sialic acids (SA), aTS allows it to circumvent this limitation by transferring α2,3-linked SA from host donor sialoconjugates to acceptor terminal β-galactopyranoses mainly found in mucin-type proteins on the parasite membrane [15]. This enzyme is also able to transfer the sialyl residue between host macromolecules [12]. Hence, TS plays a central role in *T. cruzi* pathogenesis through the modification of the surface sialylation pattern, either by sialylation (*trans*-sialidase activity) or the associated desialylation processes (sialidase activity) [12,13]. Moreover, we have recently demostrated that the iTS isoform is also involved in parasite-induced pathogenesis, probably by adhesion mechanisms [16].

Shedding TS into the bloodstream allows *T. cruzi* to exert its manipulation systemically on different host cells, such as thymocytes [17], lymphocytes [18,19], platelets [20], etc. We have also shown the differential expression of aTS and gene distribution among the different *T. cruzi* Discrete Typing Units (DTUs, DTU I-VI, as defined by genetic markers) [21,22]. Highly virulent parasite strains, included in DTU-II and VI express and shed higher amounts of aTS, inducing the worst thymus and spleen damage [17,21,23], whereas low-virulence DTU-I strains, induced minor alterations which are barely detectable *in vivo* [21,22].

Here we analyze the circulating MMP profiles in acute and chronic models of *T. cruzi* infection to better understand Chagas disease pathology development. We found that virulent parasite strains expressing high TS activity increased MMP-2 presence in the bloodstream. Therefore, we studied the modulation of systemically induced MMPs by *T. cruzi* through its shed TS. We show for the first time that TS activity is involved in MMP-2 modulation through an α2,3-linked SA specific desialylation, an effect also caused by sialidases from other pathogens, which points to a widespread pathogenic mechanism.

## 2 Material & methods

### 2.1. Ethics statement

The study was carried out in accordance with the Basel Declaration. Protocol (N° 10/2017) was approved by the Committee for Experimental Animal Care and Use of the Universidad Nacional de San Martín (UNSAM), following the recommendations of the *Guide for the Care and Use of Laboratory Animals* of the National Institute of Health (NIH).

### 2.2. Parasites

Bloodstream trypomastigotes from the *T. cruzi* RA, Tulahuen and Cvd strains and the Tulahuen-derived clone Q501/3, all high virulent parasites included in DTU-VI, were used as acute model of infection. Whereas the Ac and CA-I strains and CA-I-derived clone K-98, all low virulent parasites included in DTU-I, were used as chronic model of infection [21]. Parasites were maintained by serial passages in mice. For *in vitro* assays, culture-derived parasites from Cvd strain and the clone Q501/3 were obtained from Vero cells. Briefly, infected cells were maintained in Dulbecco’s Modified Eagle Medium (DMEM) (Gibco) with 3.7 g L^−1^ NaHCO_3_ and supplemented with 10% FBS (Gibco) at 37 °C and 5% CO_2_ atmosphere. Seven days pi (post-infection), trypomastigotes were harvested from supernatants and washed with 5% BSA in PBS for the *in vitro* assay.

### 2.3. Recombinant trans-Sialidase

His-tagged aTS [24] and iTS [14] were expressed in *Escherichia coli* BL21-DE3 and purified to homogeneity by immobilized metal affinity chromatography through Ni^2+^-charged Hi-Trap chelating columns (GE Healthcare) followed by ion-exchange chromatography (Mono Q; GE Healthcare) by an FPLC system (AKTA, Pharmacia), followed by passage through a polymyxin column (Pierce) for endotoxin depletion as described previously [17]. Finally, TS activity was confirmed by SA transfer from α2,3 sialyllactose to *d*-glucose-[^14^C]-lactose as described elsewhere [25].

### 2.4. Mice experimental design

Male 6 to 8 weeks-old BALB/c mice were infected with 50 (RA, Cvd or Q501/3), 500 (Tulahuen) or 5 × 10^4^ (Ac, CA-I and K98) bloodstream trypomastigotes by intraperitoneal route. Normal mouse serum (3% in PBS) was administered to mice of the non-infected control group (NI). Parasitemia was determined by counting in Neubauer chamber. Plasma samples from NI and infected mice were obtained at different times post-infection (pi) and frozen-stored at −80 °C for further analysis. Circulating *trans*-sialidase activity was evaluated is plasma samples from infected mice, as control of our assays [21]. Alternatively, mice were inoculated with 1 μg of recombinant aTS, iTS or BSA (Sigma) as control and 6 h post-administrations plasma samples were taken. At least six animals per group were used [25].

### 2.5. Evaluation of T. cruzi gelatinolytic activity by zymography assay

Purified bloodstream trypomastigotes, 10^7^ mL^−1^ of Tulahuen strain or 10^7^ mL^−1^ of K98 clone, were incubated during 5 h at 37 °C. After centrifugation supernatants were electrophoresed and zymography assay was performed at the same conditions described below.

### 2.6. In vitro assay

HT1080 cells were maintained in DMEM with 3.7 g L^−1^ NaHCO_3_ and supplemented with 10% FBS (Gibco) at 37 °C in a humidified environment containing 5% CO_2_. Then, 5 × 10^4^ cells per well (6 wells per treatment) were seeded in 24-well plates (Nunc). After 16 h, cells were infected with 5 × 10^5^ culture-derived trypomastigotes of the Cvd strain or the clone Q501/3 for 16 h and washed. Media were replaced 48 hours pi with FBS free media and 24 h later conditioned supernatants were collected (referred as supernatants), centrifuged and frozen-stored at −80 °C. Alternatively, cells were treated with 1 μg mL^−1^ BSA, recombinant aTS, iTS or neuraminidases from *Salmonella typhimurium* (Neu Sal) (Biolabs) or *Clostridium perfringes* (Neu Clost) (Sigma) and were processed as the infected cells. Three independent experiments were performed for each strain and neuraminidases. aTS activity was confirmed in the presence of 10 mmol L^−1^ Lactitol (Sigma), α2,3-sialyllactose or α2,6-sialyllactose (Carbosynth) as previously described [17]. For signalling inhibition assays, cells were preincubated with 10 μmol L^−1^ of Chelerythrine (LC Laboratories), U0126 or PP2 (Cell Signalling).

### 2.7. Gelatin zymography

To evaluate gelatinase activity in plasma samples, zymography was performed as described previously [26]. Briefly, protein concentration was determined by Bradford (BioRad) and equal amounts of total protein were mixed with loading buffer without a reducing agent and electrophoresed through a 7.5% polyacrylamide gel copolymerized with gelatin 1% (Sigma). An aliquot of supernatant of HT1080 cells stimulated with Phorbol Mirystate Acetate (PMA, 1 μmol L^−1^) (Sigma) was used as reference for proMMP-9, MMP-9, proMMP-2 and MMP-2 band position. Gels were immersed in rinsing buffer (50 mmol L^−1^ Tris-HCl, pH 7.5) with 2.5% Triton X-100 (PanReac) for 1 h, washed three times with rinsing buffer and incubated for 48 h in activation buffer (50 mmol L^−1^ Tris-HCl, 150 mmol L^−1^ NaCl, 10 mmol L^−1^ CaCl_2_, pH 7.5) at 37 °C. Gels were stained with Coomassie brilliant blue (Sigma). Gelatinolytic activities were detected as pale bands against the dark blue background. Band intensities were quantified using ImageJ software and expressed as Arbitrary Units (AU). During electrophoresis, Sodium dodecyl sulfate (SDS) caused latent MMPs to become active and the gelatinolytic activity distinguished on the basis of their molecular weight [27]. To confirm the gelatinolytic proteinase activities either 1,10-phenanthroline (1 mmol L^−1^, Sigma), Ethylenediaminetetraacetic (EDTA) (10 mmol L^−1^, Sigma) or Phenylmethyl Sulfonyl Fluoride (PMSF) (1 mmol L^−1^, USB) was added to the incubation buffer (Supp. Fig. 1) [28,29].

### 2.8. Western blot

Equal amounts of plasma protein were electrophoresed through a 7.5% polyacrylamide gel under reducing conditions, and transferred with 25 mol L^−1^ Trizma base; 92 mmol L^−1^ glycine, 20% v/v methanol, pH 8.3 onto polyvinylidene fluoride membranes (GE). Membranes were blocked with 20 mmol L^−1^ Tris—HCl, 150 mmol L^−1^ NaCl, pH 7.4, non-fat milk 5%, incubated with anti-MMP-2 antibody (Santa Cruz Biotechnology), incubated with rabbit Horseradish peroxidase (HRP)-IgGs anti-goat antibodies (Dako.) They were revealed using Supersignal CL-HRP Substrate System (Pierce), chemiluminescence was recorded with the ImageQuant equipment (GE), bands were quantified using ImageJ software.

### 2.9. Statistics

Differences between experimental groups were analyzed by one-way ANOVA in conjunction with Tukey’s test. Differences were considered statistically significant when *p* < 0.05. Data obtained from MMP-2, MMP-9 and its latent forms activity quantification were normalized to 1 (with 1 being the band intensity of the reference) and expressed as means ± SEM.

For the *in vivo* infection procedures, data obtained at day 15 pi from the infection with highly virulence strains were compared against data obtained for NI mice and for low-virulence strains at each time point (*i.e.* RA infection at day 15 pi *vs.* Ac infection at day 15 pi, RA infection at day 15 pi *vs.* Ac infection at day 30 pi, etc). Repeated measures ANOVA and Dunett’s multiple comparisons were performed to compare data from each strain of low-virulence treatment against data from NI mice. For graphical simplification, grouped statistics are shown. Tests were performed using Graph-Pad Prism version 7.00 for Windows, GraphPad Software, La Jolla, California USA.

## 3. Results

### 3.1. Trypanosoma cruzi strains induce alterations in plasmatic MMPs activity

**Fig. 1.**
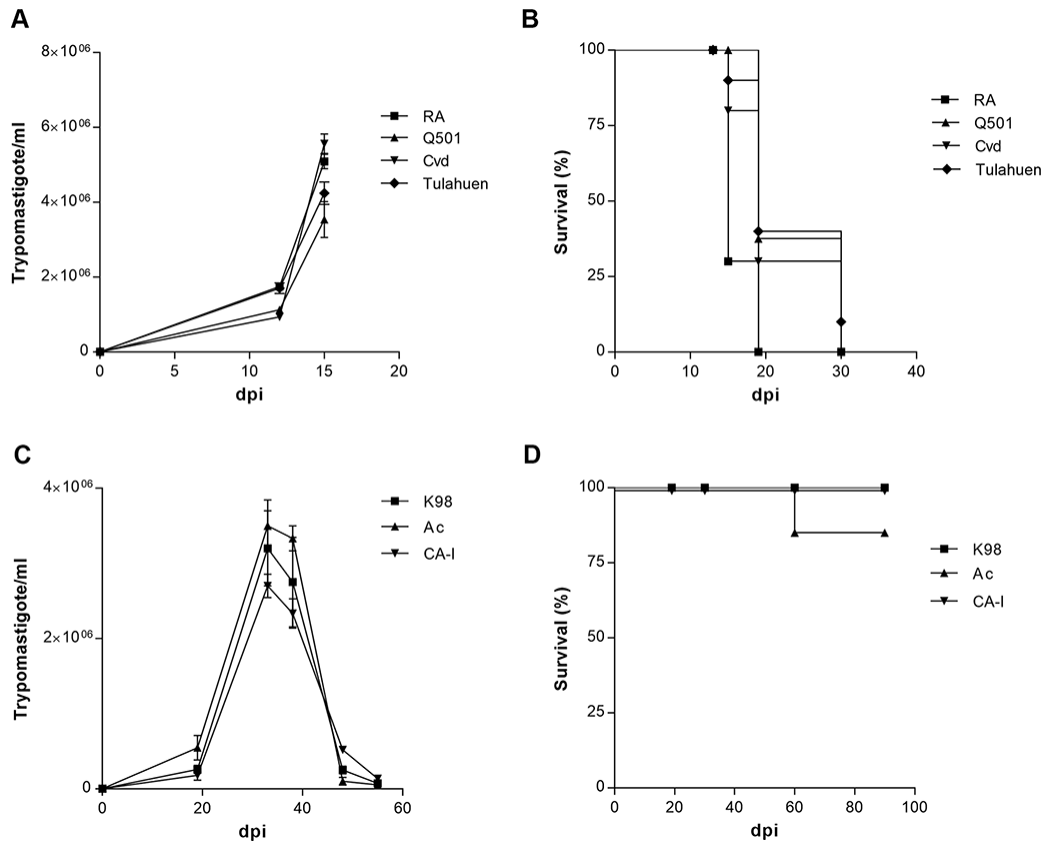
Parasitemia (A, C) and survival (B, D) of highly virulent strains (A, B) and low-virulence strains (C, D).

**Fig. 2.**
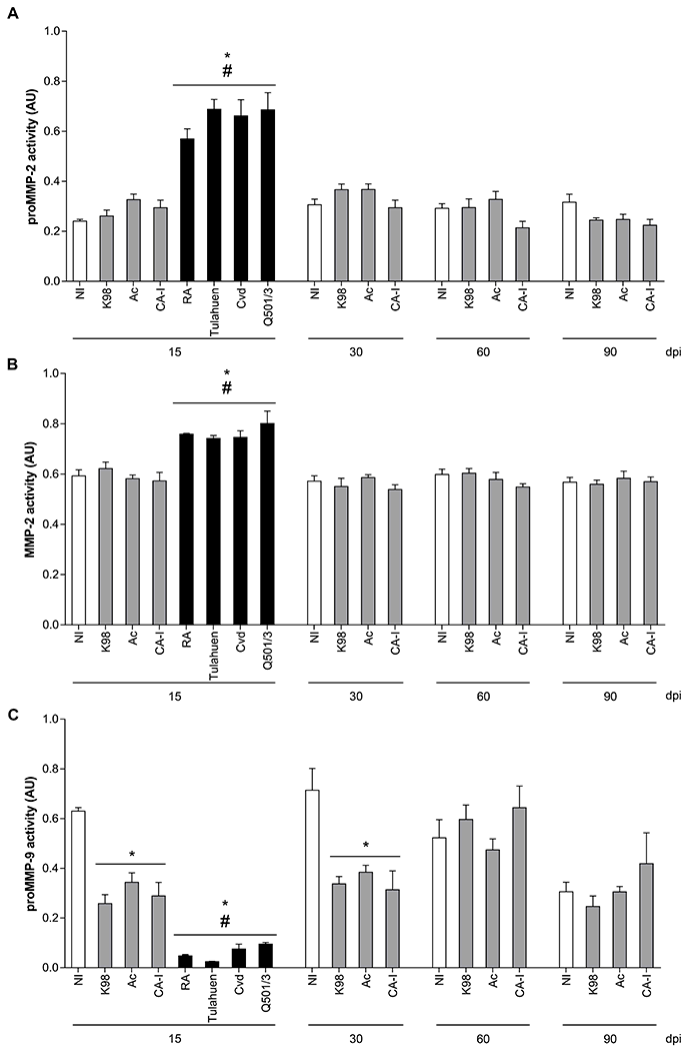
Quantification of circulating MMPs in infected mice. (A) proMMP-2, (B) MMP-2 and (C) proMMP-9 gelatinolytic activity from non-infected mice (NI, white bars), mice infected with low-virulence parasites (gray bars) and highly virulent parasites (black bars) at indicated dpi. ANOVA and Dunett’s tests were used for multiple comparisons (highly virulent strains vs low-virulence strains/NI, low-virulence strains vs NI, see Materials and Methods). * 0.0001 < p < 0.05 vs NI; # 0.0001 < p < 0.05 *vs.* low virulence strains.

To analyze the expression of MMPs during *T. cruzi* infection, we used acute and chronic *T. cruzi* infection models established in our laboratory [21]. Highly virulent strains (HVS) RA, Tulahuen, Cvd, and clone Q501/3 (all from DTU-VI) induce lethally acute infections, whereas low-virulence strains (LVS) CA-I, Ac and clone K-98 (all from DTU-I), induce chronic infections (Fig. 1). In the acute model the plasma samples were obtained at day 15 pi, while in the chronic model were taken either at 15 dpi, during the parasitemia peak (30 dpi) and after 60 and 90 dpi. The active and proMMP-2 forms showed increased activity in mice infected with HVS strains compared to non-infected mice, no significant differences were observed when infecting with LVS (Fig. 2 A, B). On the other hand, proMMP-9 activity was significantly reduced during the acute phase of both murine models (at 15 and 30 dpi) (Fig. 2C), (Supplementary Fig. 2).

We have analyzed by zymography the presence of metallopeptidase secreted by *T. cruzi* trypomastigotes, in supernatants of shedding of Tulahuen and K98 strains. No degradative bands were observed (Supplementary Fig 3).

Plasma samples from HVS infected mice, obtained at 15 dpi, and from LVS infected mice obtained at 30 dpi, were also analyzed by Western blot. Samples from mice infected with HVS showed higher reactivity with anti-MMP2 antibody, respect to samples from mice infected with LVS and the control group (Supplementary Fig. 4A). An expected 62 KDa higher reactivity bands were observed in samples from mice infected with HVS (Supplementary Fig. 4B).

### 3.2. trans-Sialidase activity increases plasmatic proMMP-2 in vivo

To understand the mechanisms involved in MMP activity modulation by *T. cruzi*, and considering that HVS express and shed higher amounts of the virulence factor TS [21], we evaluated TS activity as a possible MMPs modulator. In this study TS activity in plasma from infected mice, were similar to those communicated by Risso et al., [21].

In Supplementary Table 1 TS activity values obtained are shown.

**Fig. 3.**
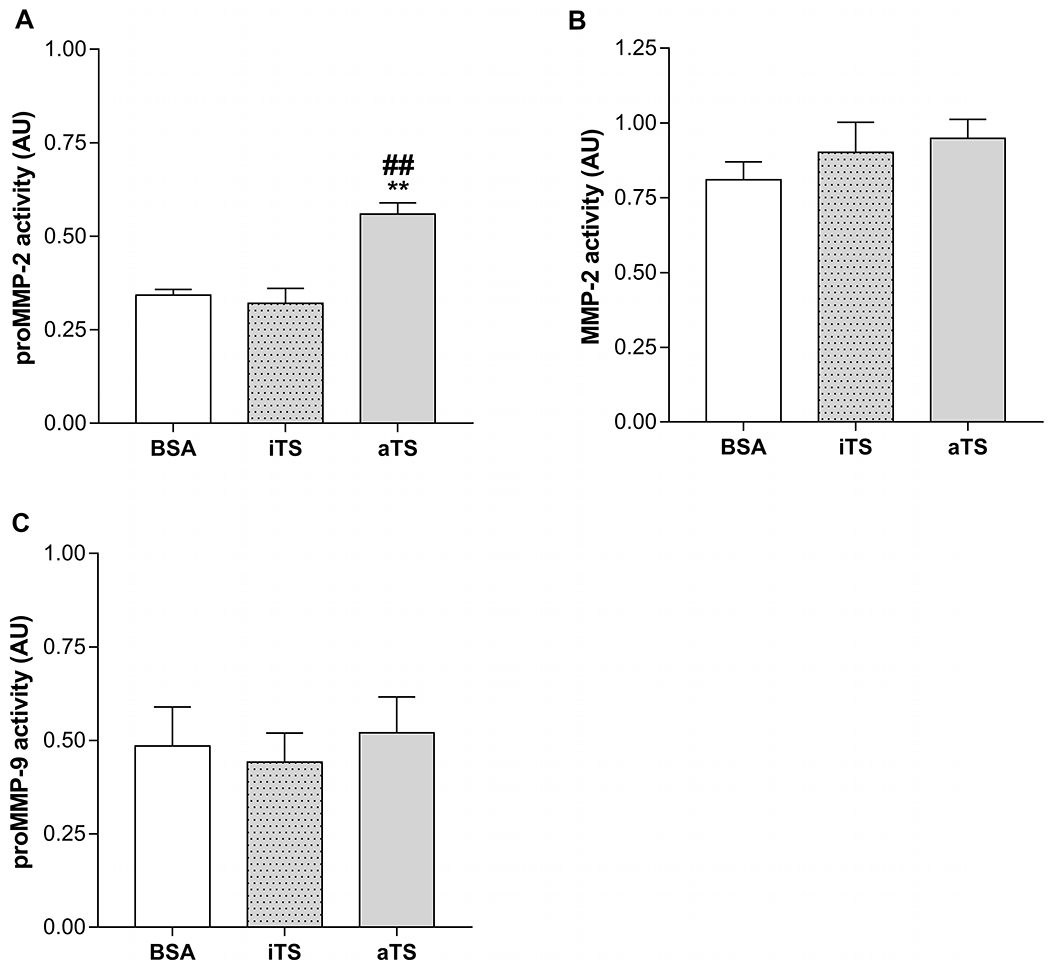
Quantification of circulating MMPs after trans-sialidase treatment. (A) proMMP-2, (B) MMP-2 and (C) proMMP-9 gelatinolytic activity from mice treated with BSA, iTS or aTS. Samples were collected at 6 h after treatment administration. **p < 0.01 vs BSA ##p < 0.01 vs iTS.

Naive mice were administered the active (aTS) or inactive (iTS) recombinant protein or BSA, and blood samples were taken 6 h later. Remarkably, proMMP-2 plasma activity from mice receiving aTS was significantly higher than in mice treated with iTS or control (Fig. 3A, Supplementary Fig. 5). MMP-2 and MMP-9 activity were similar between aTS, iTS and control mice (Fig. 3B and C).

### 3.3. T. cruzi infection modulates MMP-2 and MMP-9 activity in human cells

**Fig. 4.**
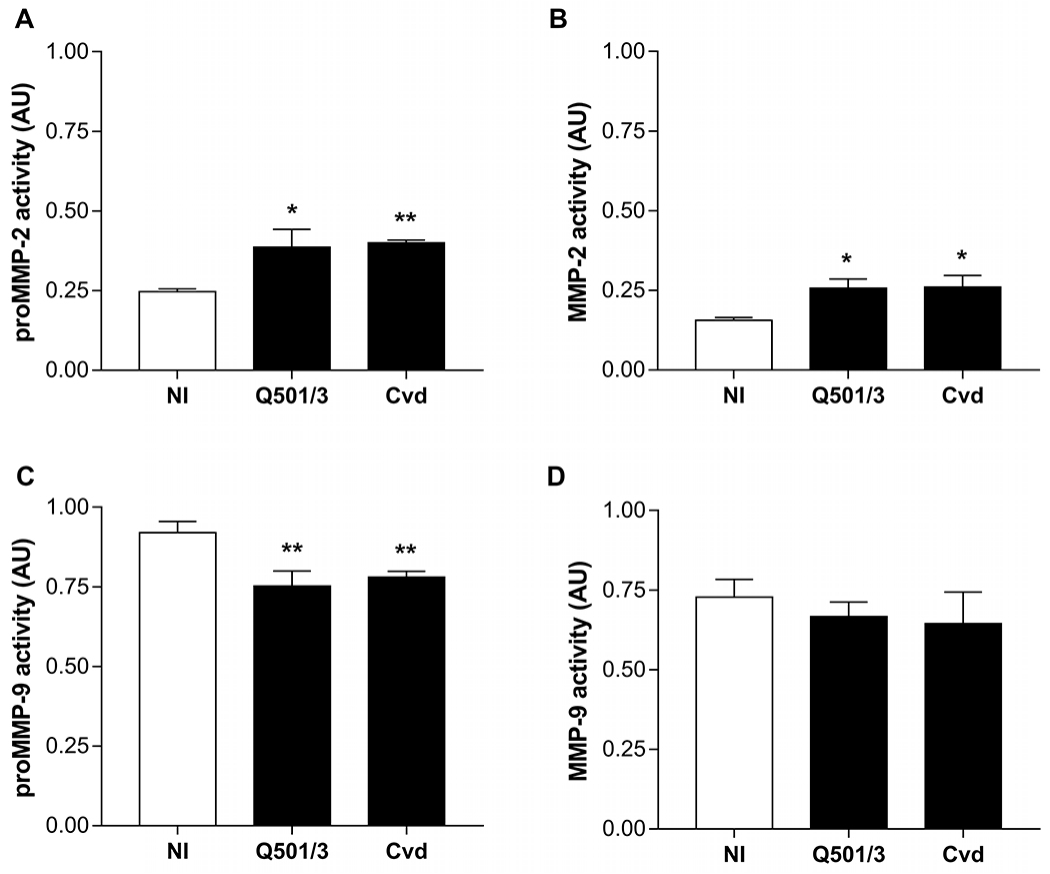
Quantification of MMPs in HT1080 infected cells. (A) proMMP-2, (B) MMP-2 (C) proMMP-9 and (D) MMP-9 gelatinolytic activity were evaluated in supernatant from HT1080 cells infected with Cvd or Q501/3 parasites. Samples were collected at 2 dpi. *p < 0.05; **p < 0.01.

Modulation of MMP-2 and MMP-9 activity by *T. cruzi* was evaluated using HT1080 fibrosarcoma human cells that secrete these MMPs [30]. They were infected either with the Cvd strain or the Q501/3 clone parasites (both HVS), and secreted MMP gelatinolytic activity was measured at day 2 pi. Supernatants from infected cells showed zymography patterns similar to those from *in vivo* assays. Indeed, the enhancement of MMP-2, in both active and pro-enzyme forms, together with the reduction of proMMP-9 activity closely mimicked the *in vivo* findings (Fig. 4A–C). Moreover, we observed no alterations in MMP-9 levels in culture supernatant (Fig. 4D).

### 3.4. TS neuraminidase activity modulates MMP-2 in human cells

**Fig. 5.**
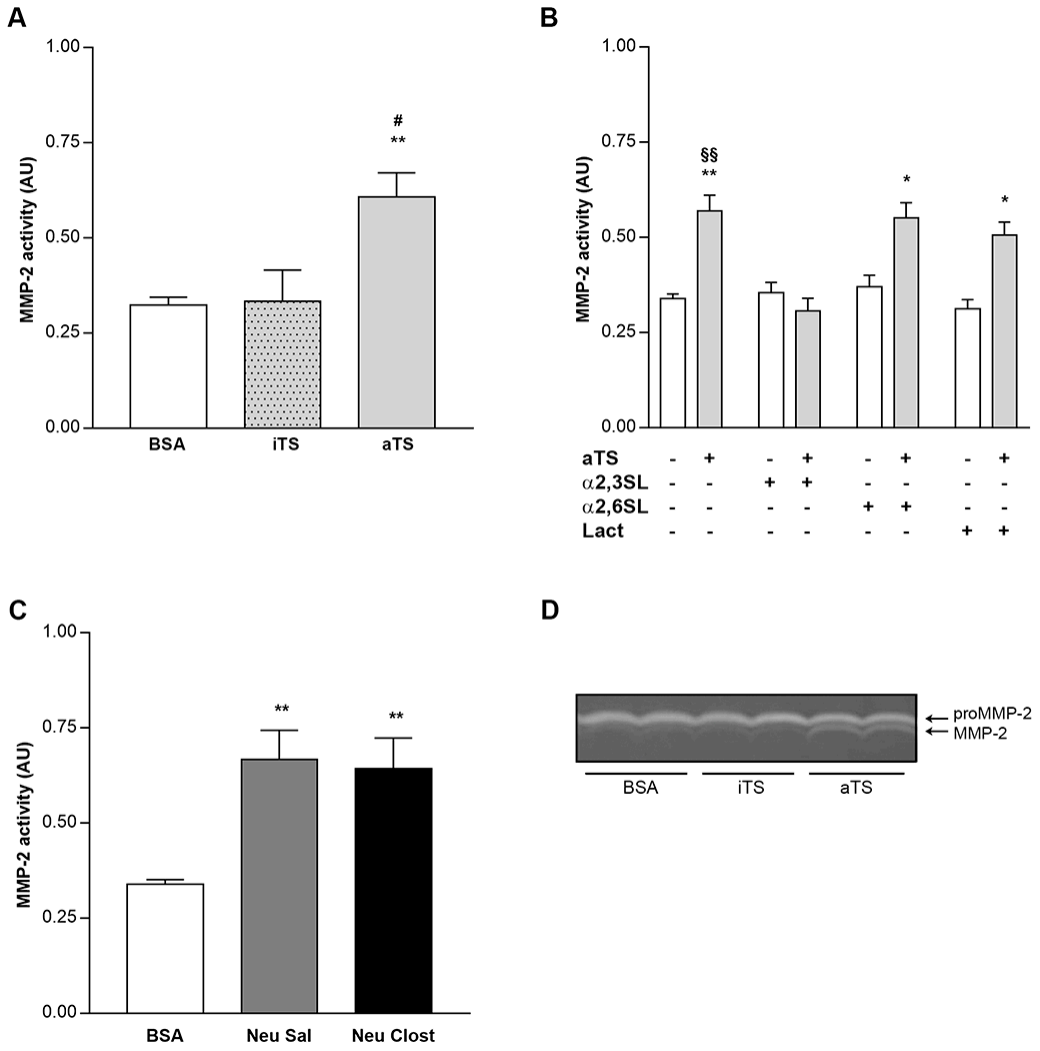
Quantification of MMP-2 activity in supernatant of HT1080 cells treated with: (A) BSA, iTS or aTS (B) aTS in presence of α 2,3SL (α 2,3 sialyllactose), α 2,6SL (α 2,6 sialyllactose) or Lac (Lactitol). (C) BSA, Neu Sal or Neu Clost (D) representative zymogram obtained from supernatant of cells treated with BSA, iTS or aTS. Arrows indicate the latent (72 KDa) and active (62 KDa) forms of MMP-2. *p < 0.05; **p < 0.01 *vs.* their respective controls; #p < 0.05 vs iTS; §§p < 0.01 vs aTS + α 2,3SL.

To evaluate TS activity as a possible MMP regulator in a human cell model, we incubated cells with recombinant aTS, iTS or BSA overnight. MMP-2 activity in supernatants from aTS-incubated HT1080 cells was higher than those from cells treated with iTS or BSA (Fig. 5A, D), supporting that TS catalysis is required for the induction of MMP-2 activity. In order to discriminate which TS activity (sialidase or *trans*-sialidase) was mediating the increase of MMP-2 activity, HT1080 cells were co-incubated with aTS in presence of lactitol (a preferential SA acceptor that enhances TS-neuraminidase activity on the cell surface), α2,3 sialyllactose (α2,3SL, a molecule that acts as preferential donor of SA for TS transfer activity) [17] or α2,6 sialyllactose as a control (α2,6SL, SA in a configuration not recognized by TS). Supernatants from cells co-incubated with aTS/lactitol or aTS/α2,6SL showed higher MMP-2 activity than supernatants from aTS/α2,3SL (Fig. 5B, Supplementary Fig 6B). Thus, increased MMP-2 activity is related to aTS neuraminidase activity.

Several pathogens possess neuraminidases that contribute to the establishment of the infection or to pathogenesis [31,32]. To determine if a bacterial neuraminidase activity can mimic the effect of *T. cruzi* sialidase activity on MMP modulation, we co-incubated HT1080 cells with commercially available purified neuraminidases from *Salmonella typhimurium* (Neu Sal., cleaves SA in α2,3 configuration) and *Clostridum perfringens* (Neu Clost., cleaves SA in α2,3, α2,6 and α2,8 configurations). In accordance with our previous observations, Neu Sal and Neu Clost, resembled the increased MMP-2 expression induced by aTS (Fig. 5C, Supplementary Fig. 6A,C).

### 3.5. TS neuraminidase activity triggers PKC/MEK/ERK signaling pathway

**Fig. 6.**
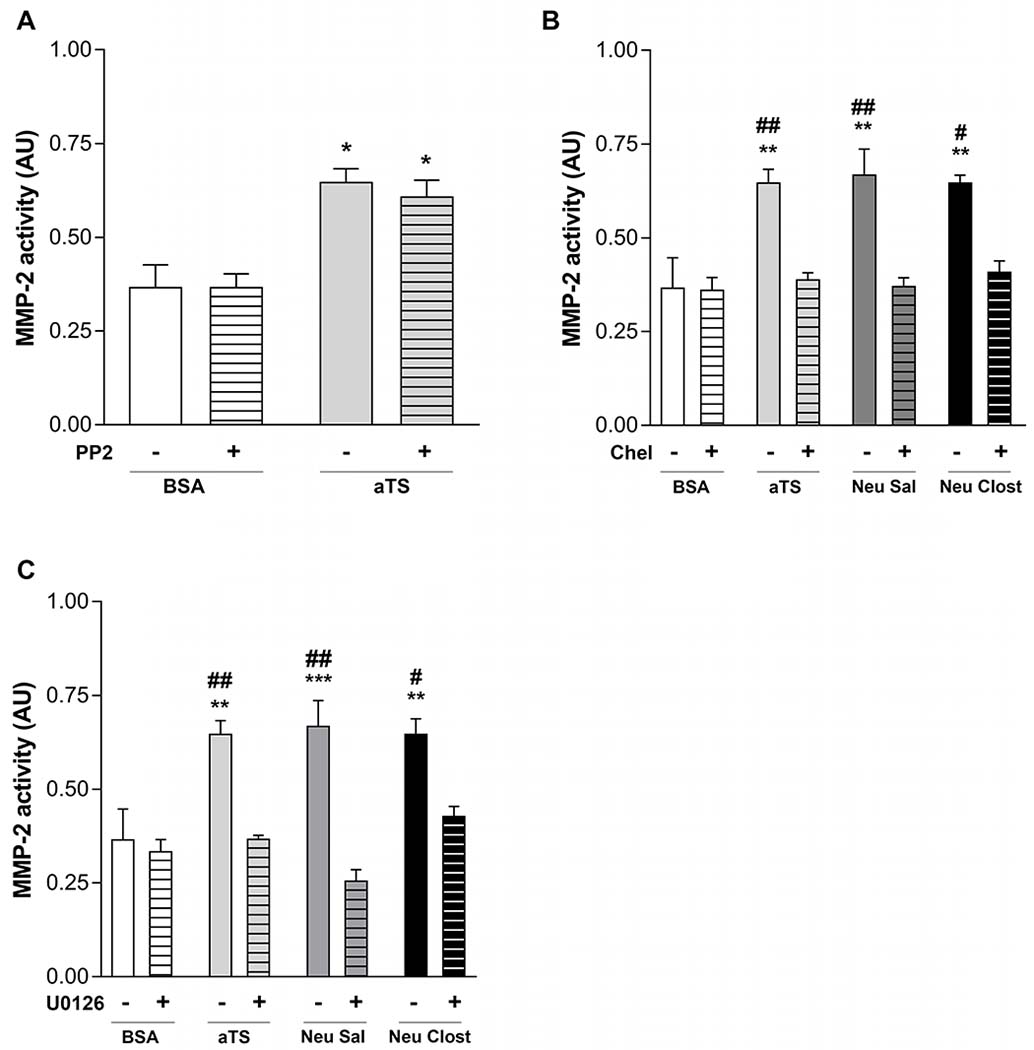
Quantification of MMP-2 activity in supernatant from HT1080 cells treated with: aTS, Neu Sal (*Salmonella typhimurium* neuraminidase), Neu Clost (*Clostridium perfringens* neuraminidase) or BSA and pretreated or not with (A) PP2; (B) Chelerytrine or (C) U0126. *p < 0.05; **p < 0.01 vs BSA; #p < 0.01; ##p < 0.01 vs the correspondent enzyme + inhibitor.

To elucidate the cell signaling mechanisms involved in aTS-induced MMP-2 activity upregulation, HT1080 cells were co-incubated with aTS, Neu Sal or Neu Clost, in presence or absence of PP2 (SRC family inhibitor), Chelerythrine (Chel., PKC inhibitor) or U0126 (MEK1/2 kinase inhibitor). While PP2 did not display significant differences (Fig. 6A), MMP-2 activity upregulation was lost when cells were treated with Chel or U0126 (Fig. 6 B, C), which shows that the PKC/MEK/ERK pathway mediates this regulation.

## 4. Discussion

MMPs are involved in numerous homeostatic and physiological processes, such as cell migration, angiogenesis, immune response and innate immunity [2]. Their dysregulation can lead to the development of several pathologies [33], including those associated with infectious diseases [34–36]. In sleeping sickness, for instance, an increase in MMP-2 and MMP-9 favors leukocyte penetration into brain parenchyma, and parasite load and inflammation intensity in canine leishmaniosis [37,38]. Furthermore, in helminth infections (neurocysticercosis) differential expression of circulating MMP-9 enables discrimination of asymptomatic and symptomatic patients [39]. Thus, their role in infectious processes have placed them as therapeutic targets [40].

Particularly in Chagas Disease, neutrophils and monocytes show differential expression of MMP-2 and MMP-9 in the bloodstream [41]. Also the relevance of infected cardiac macrophages in driving to a profibrotic profile through MMP2/MMP9-mediated TGF-β activation was highlighted [42]. The association between the clinical form and the plasma activity of MMP-2 and MMP-9 suggests both gelatinases could be markers for status and prognosis of clinical evolution [5,43–46].

In this study, we tested *T. cruzi* strains that exhibit high or low virulence, typified as DTU VI and I respectively, and found that they modulate MMPs secretion differently. HVS increased plasmatic MMP-2 activity, while all strains decreased proMMP-9 levels during the acute phase, being HVS the major dimmers. Other authors have also described alterations in MMP-2 and MMP-9 profiles during the acute phase in different experimental models [10,11,47].

In our study we included, as control, the evaluation of metallopeptidases secreted by trypomastigotes. Cuevas et al. have reported that the Tcgp63-I, is expressed in the different parasite stages, but a very low metalloprotease activity in epimastigotes extracts was observed by zymography, using 250 × 10^6^ parasites [48]. In agreement, Rebello et al. also observed gp63 gelatinolytic activity in epimastigotes stage extracts [49]. It is important to note, that the maximum number of circulating parasites of samples that we analyzed, were 4 × 10^6^ mL^−1^ (Cvd strain) and 3,5 × 10^6^mL^−1^ (Ac strain), (Fig. 1). In addition, the absence of gelatinolytic activity in samples from shedding of trypomastigotes, let us propose that the MMPs profile observed in circulation were induced in mice by *T. cruzi*.

The evaluation by Western blot of circulating MMPs in infected mice, showed the same MMP-2 prolife as the described by zymography. During the acute phase, HVS induced higher MMP-2 level than LVS or that found in non-infected mice. Then during the mice infection, a similar upregulation of MMP-2 fraction was observed by different methods of analysis.

Both MMP-9 protein and activity are tightly regulated so they are kept at physiological amounts [50]. Of interest, the formation of MMP-9 complex with α2-macroglobulin, its main inhibitor in plasma, and the binding of this complex to low density lipoprotein receptor-related protein to be internalized, could be mentioned [51,52]. Concerning *T. cruzi* infection, both α2-macroglobulin and low density lipoprotein receptor are known to be increased by the parasite [53,54]. Nogueira de Melo et al. [47] also observed a decrease in proMMP-9 secretion without detection of its active in supernatants of primary cell culture infected hepatocytes. In our study, the decrease of proMMP-9 activity in infected mice plasma might result from its higher activation, leading to an increase in active MMP-9 which in turn might be sequestered by forming a complex with α2-macroglobulin [55]. The different levels of regulation, might also be modulated by parasite molecules to benefit the infection and its persistence in the host. In other models, such as in *Staphylococcus aureus* infection, Staphylococcal SuperantigenLike Protein 5, inhibits MMP-9 activity by binding to pro-MMP-9, in human neutrophils [56]. It has also been described the ability of LPS to modulate mRNA levels of MMP-9 in mice organs [57]. Besides, enhanced MMP-9 circulating levels have been described in an endotoxemia model [58]. Cytomegalovirus has also shown the ability to reduce MMP-9 activity in human macrophages [59]. All these evidence shows that the modulation of MMP-9 by *T. cruzi* seems to be dependent of many factors including the interaction of different parasite strains with a heterogenous and complex host scenario. During the acute phase different alterations are observed [60] and several cytokines are involved in assembling an effective immune response, the pro-inflammatory environment induced requires to be under a tight control to avoid host damage [61,62]. Interestingly, TNF-α, IFN-γ and IL-1β are tightly linked to gelatinase regulation at different levels, either enhancing or inhibiting their expression or activation, and can also act in concert with them [50,63–65]. The inflammatory process and the different tissues affected may be involved in the MMPs alterations induced by *T. cruzi.* Considering, our *in vivo* assays, we related the differential modulation of MMPs activity to parasite virulence and to different *T. cruzi* DTUs. This association could explain discrepancies between MMP profile and clinical status, observed in some publications, which used samples from different endemic areas where different DTUs predominate because they were infected with different parasite strains [66–68].

To further analyze the molecules involved in MMPs modulation and considering that the sialylation state of the cell surface has been related to MMP-2 and MMP-9 expression/activity modulation [69], we decided to explore if TS activity was involved. It is important to note that HVS, that express/shed high amount of TS activity, induced *in vitro* the same MMP profile observed *in vivo*.

TS contains a repetitive region responsible for extending the permanence of the enzyme in the bloodstream [12] favoring the generation of surface sialylation pattern disorders in host cells [17,18,20,70]. Here, we found that aTS induced an increase in plasmatic proMMP-2, resembling what is observed during HVS *T. cruzi* infection. This might be associated with the mechanisms of MMP-2 activation by MT1-MMP (MMP-14) [71], a glycoprotein containing α2,3-linked SA necessary for cell surface MMP-2 activation [72,73]. Thus, we cannot discard MMP-14 as a potential TS target. However, an increase in plasmatic proMMP-2 translates into a broader MMP-2 pool. Although iTS is involved in immunopathology and virulent parasite phenotype during experimental infections [13,16,70], it had no effect on MMP activity induction either *in vivo* or *in vitro.* Because both TS isoforms only differ in Tyr_342_His and are produced and purified through the same protocols, iTS is the best control to test the relevance of the enzymatic activity in all assays involving aTS. Then, in addition to the thrombocytopenia induced by bloodstream TS sialidase activity, it is also involved in the modulation of MMP-2. TS has to face many proteins in the blood or on the surface of cells, but it appears that the enzyme can use as an acceptor (and probably as donors) of the sialyl residue a selected group of glycoconjugates [74], showed that the enzyme preferentially attacks some proteins on the surface of the target cell, but when the cell breaks, many others become available. Therefore, it appears that the enzyme does not attack any sialylated protein when it is in its real context (for example, not in a solution, but on the surface of a cell), but has a preferential action that could be due to size or exposure of the target glycoconjugated as suggested by Muiá et al., although no other specificity can be disregarded [74]. In addition, TS is able to attack both Neu5Ac (N-acetylNeuraminic acid) and Neu5Gc (N-glicolylneuraminic acid) residues, that are the most abundant sialic acids present in mammals. Humans (and other primates) are only able to synthesize Neu5Ac although they can incorporate Neu5Gc from the diet [75].

As mentioned before, TS either acts as sialyl-transferase or as a sialidase [12]. To establish which of these TS activities are associated with the induction of MMP-2 activity secretion in human cells, we co-incubated cells with aTS and lactitol. This competitive substrate leaves the hydrolase activity undisturbed, while preventing SA transference to endogenous acceptors [17]. Lactitol failed to block the increase of MMP-2 activity. Moreover, when cells were incubated with aTS in the presence of α2,3-sialyllactose, a classical SA donor in TS-catalyzed reactions, the increase in MMP-2 was not observed, meaning that TS hydrolyzes SA from the donor instead of from cell-surface molecules. Taken together, these results show that cell surface desialylation by TS induces MMP-2 activity in HT1080 cells (Fig. 5B). Further, bacterial neuraminidases with SA a2,3 cleavage ability induced increased MMP-2 activity, suggesting that this phenomenon might not be restricted to *T. cruzi* infection only. Interestingly, while both bacterial neuraminidase and *T. cruzi* sialidase activate only MMP-2, the mammalian sialidase Neu-3, specific for SA α2,3 recognition observed in human prostate cancer models [69], is able to modulate both MMP-2 and MMP-9.

Neuraminidases are virulence factors from several bacteria, viruses and parasites, involved in the induction of diverse pathogenic mechanisms associated to their life cycles, and present different substrate specificity, SA at α2,3, α2,6, α2,8 [76–79]. In addition to *Salmonella* sp and *Clostridium* sp., there are many bacteria such as *Streptococcus pneumoniae, Vibrio cholerae, Gardenerella vaginalis*, and virus such as Influenza [2,80–83] that express sialidases able to remove α2,3 SA. Our findings show that neuraminidases regulate MMPs, a mechanism that can apply to the wide diversity of neuraminidase-expressing pathogens, which in turn can cause huge local or systemic disorders depending on their biological context.

The sialylated/desialylated state of receptors has been linked to cell signaling [84,85]. To elucidate the signaling mechanisms in TS-induced MMP-2 upregulation, aTS and other neuraminidases were evaluated in combination with inhibitors of different cell signaling pathways regulating MMPs [86–88]. While Src inhibitor PP2, had no effect, Chelerythrine and U0126, PKC and MEK1/MEK2 inhibitors respectively, abolished the TS-mediated increase in MMP-2 activity, supporting that the PKC/MEK/ERK pathway might be responsible for this upregulation.

### 4.1. Conclusions

In this study we described a novel pathogenic mechanism of TS neuraminidase activity: the upregulation of MMP-2. This new strategy, that depends on sialic acid removal, can be shared by different pathogens like bacteria and virus, revealing an important point of regulation of MMPs network by pathogens.

## Supplementary Material

7

8

9

10

11

12

13
