# Supplementary figures and images for: Sialic acid removal by trans-sialidase modulates MMP-2 activity during Trypanosoma cruzi infection
Source: Biochimie. Author manuscript; Available in PMC 2021 Jul 1. (PMC8187320; doi:10.1016/j.biochi.2021.04.005)

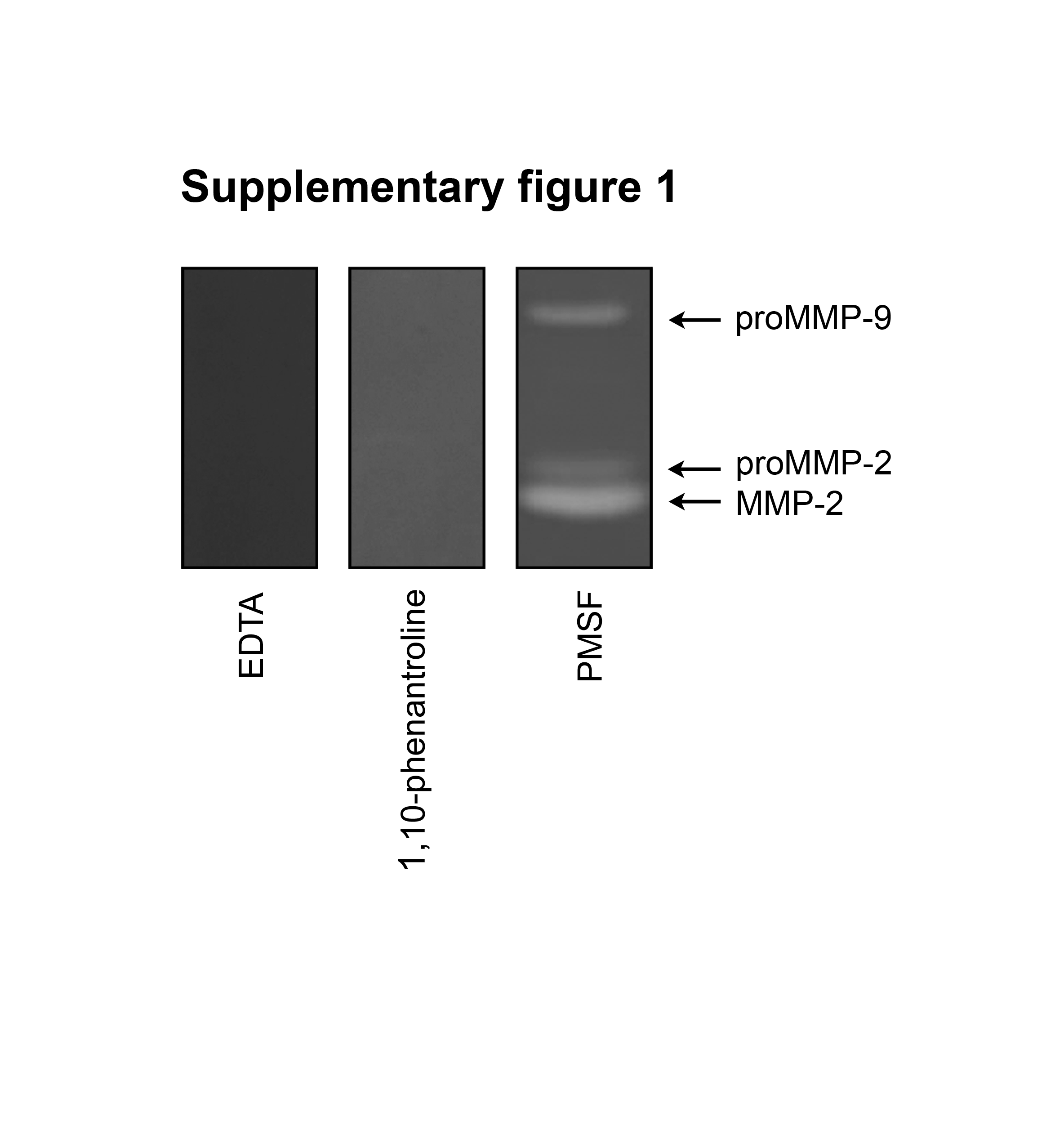

Supplement: 7 [file NIHMS1701144-supplement-7.tif]

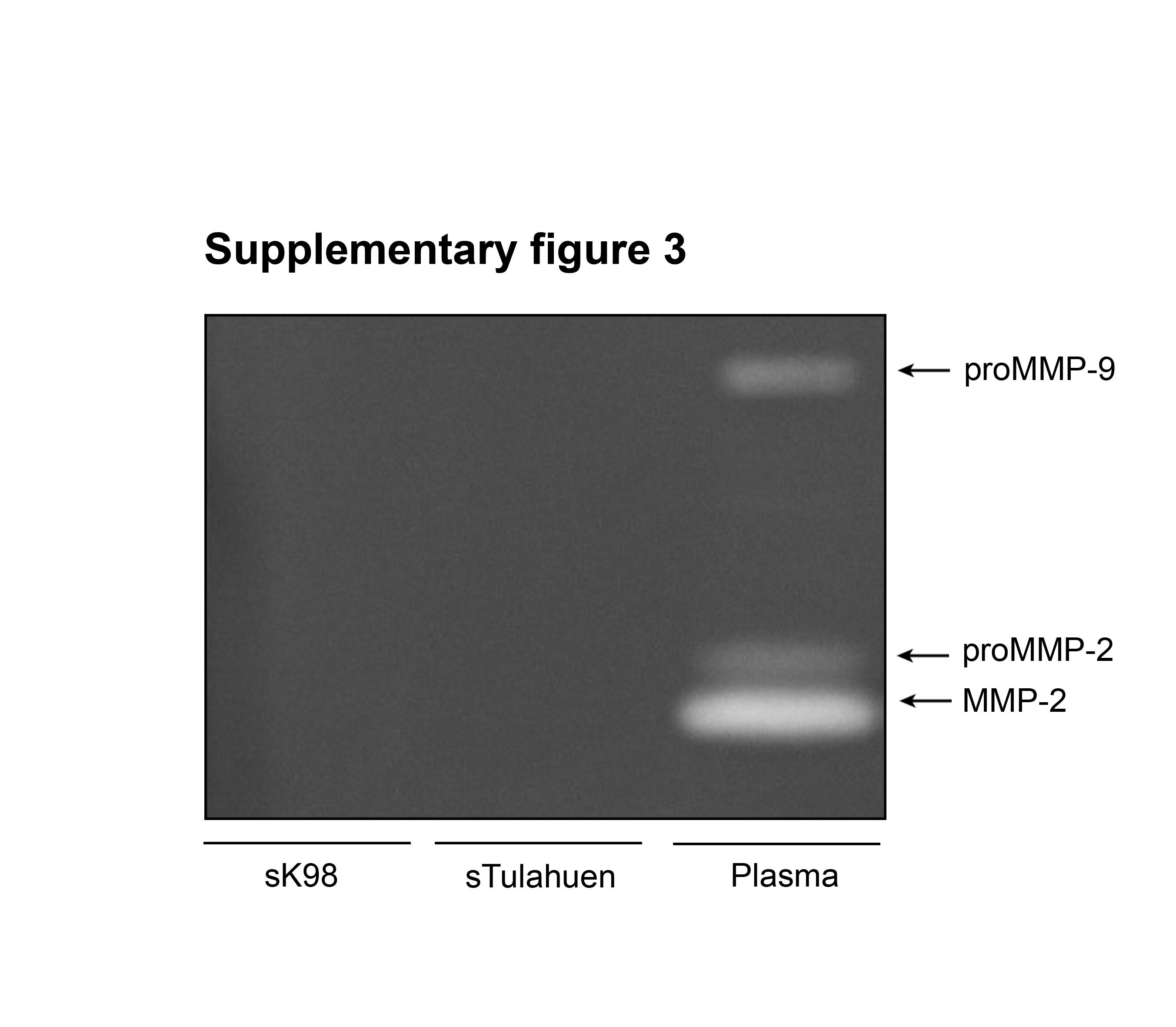

Supplement: 9 [file NIHMS1701144-supplement-9.tif]

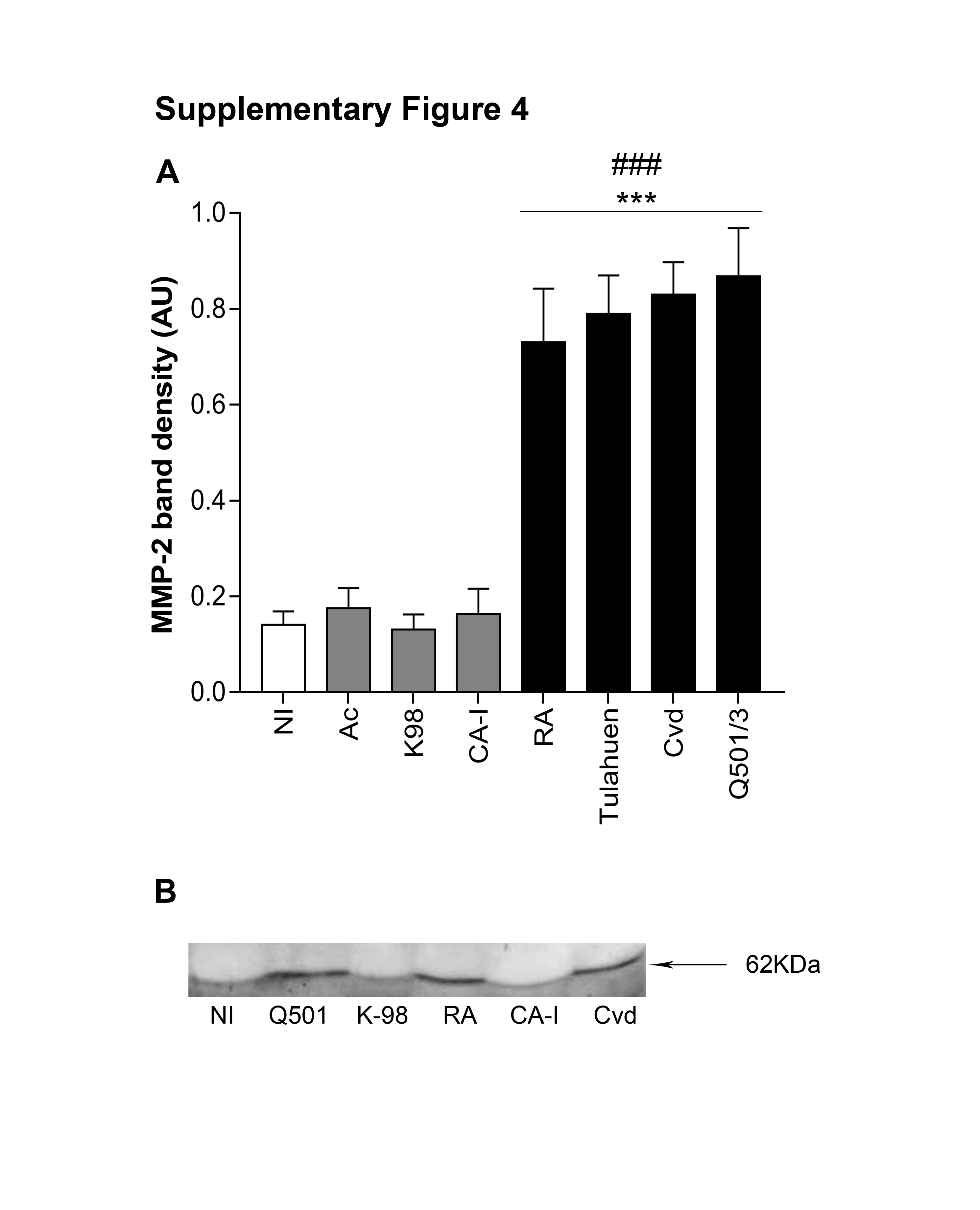

Supplement: 10 [file NIHMS1701144-supplement-10.tif]

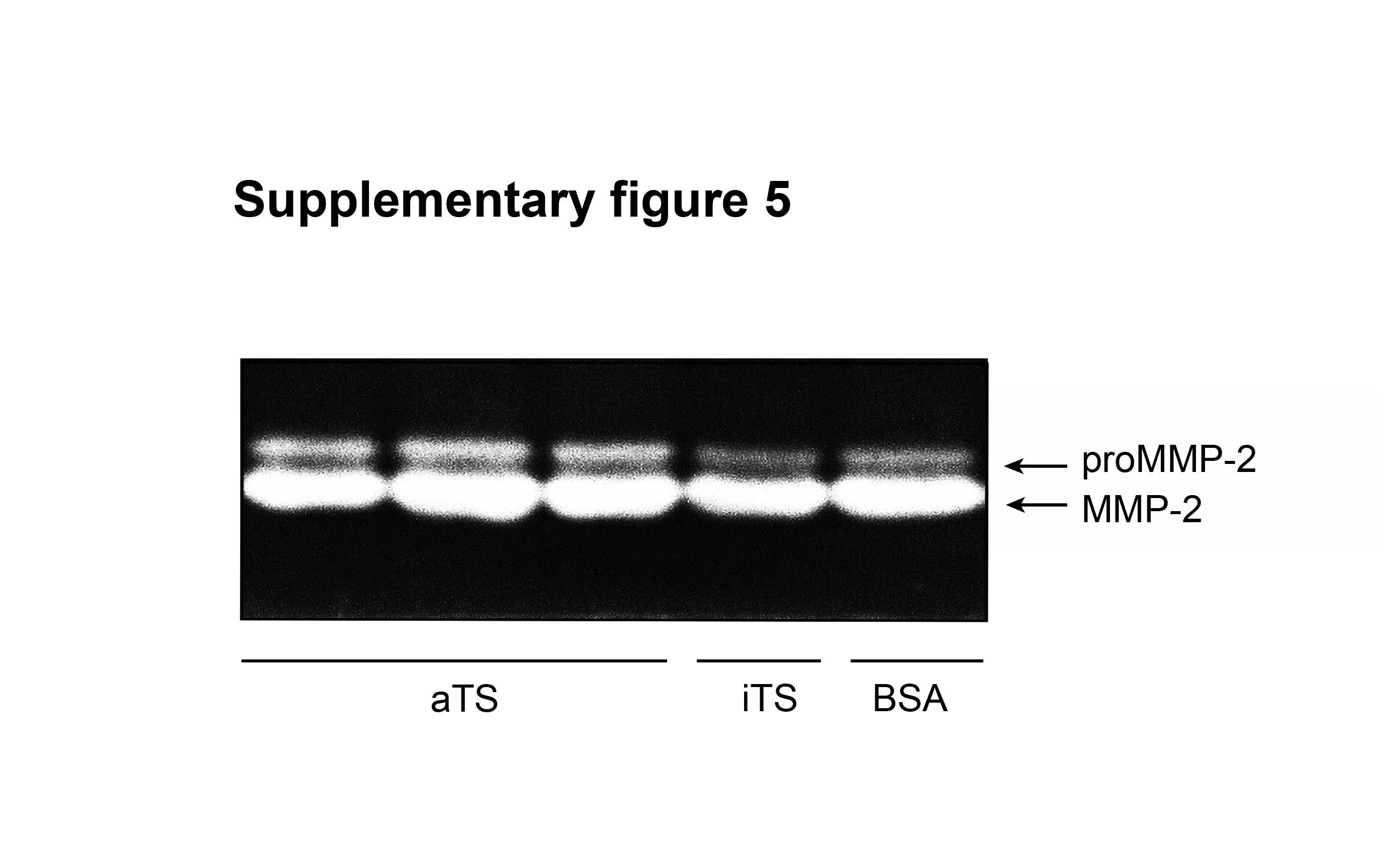

Supplement: 11 [file NIHMS1701144-supplement-11.tif]

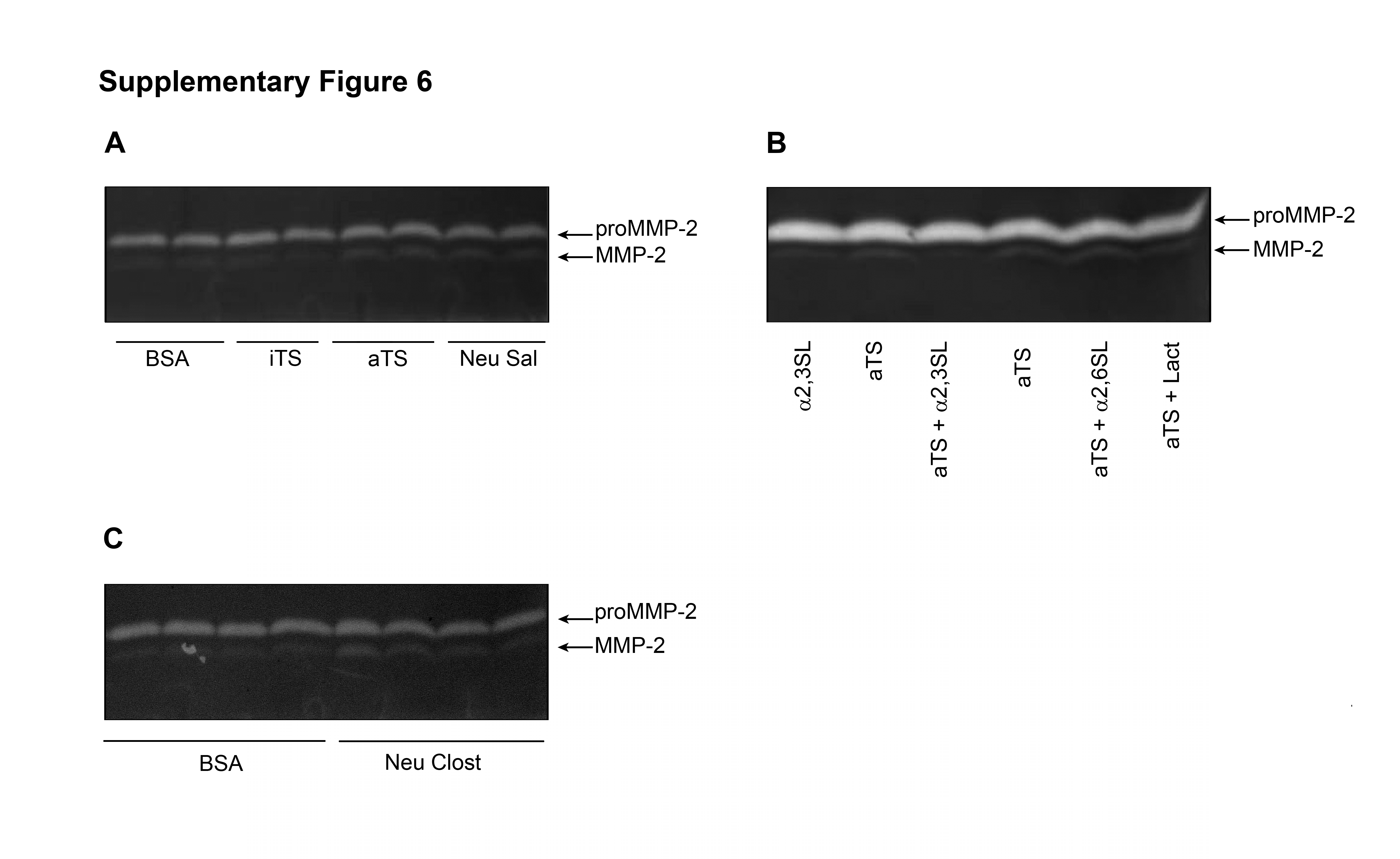

Supplement: 12 [file NIHMS1701144-supplement-12.tif]
