# Supplementary material for: Sialic acid removal by trans-sialidase modulates MMP-2 activity during Trypanosoma cruzi infection
Source: Biochimie. Author manuscript; Available in PMC 2021 Jul 1. (PMC8187320; doi:10.1016/j.biochi.2021.04.005)
Supplement: 13 [file NIHMS1701144-supplement-13.docx]

| ***T. cruzi*** | **TS activity (cpm)** |
| --- | --- |
| Tulahuen | 512.3 ± 172.4 |
| RA | 577.3 ± 126.3 |
| Cvd | 622.8 ± 51.0 |
| Q501 | 609.1 ± 112.0 |
| K98 | 11.5 ± 9.2 |
| Ac | 30.7 ± 12.9 |
| CA-I | 17.6 ± 5.5 |

Supp Table 1
